# Desmosis Coli − a Case Report and Review of The Literature

**DOI:** 10.34763/devperiodmed.20172104.390392

**Published:** 2018-01-02

**Authors:** Jayesh Desale, Hemanshi Shah, Vikrant Kumbhar, Gursev Sandlas

**Affiliations:** 1 Dept of Paediatric Surgery, TNMC & BYL Nair Hospital, Mumbai Central, Mumbai, Maharashtra. India

**Keywords:** desmosis coli, constipation, children

## Abstract

Desmosis coli is a rare pathology presenting as slow transit constipation. In this case we would like to discuss the presentation and management of desmosis coli. A 14-month-old female hailing from western India with a history of chronic constipation presented with acute massive abdominal distension and vomiting. At laparotomy, a hugely dilated transverse and sigmoid colon with a transition zone at the lower sigmoid was found. A transverse stoma was done after taking multiple seromuscular biopsies. The patient underwent re-exploration on day 14 because of the non-functioning of the stoma and a fixed bowel loop. The histopathology report was suggestive of normal ganglion cells. Unfortunately, the stoma continued not to function. A dye study showed dye in the colon after 24 hours ruling out any anatomical obstruction. Histopathology slides were reviewed multiple times and reported lack of connective tissue of the colonic wall leading to the diagnosis of desmosis coli. The patient was started on gradual feeds and pro-kinetics and over the next 2 weeks the stoma started functioning slowly. Desmosis coli is a rare cause of constipation which should be suspected in cases where aganglionosis has been ruled out and the constipation is refractory to conventional therapy.

## Introduction

Constipation is a common problem in children. Desmosis coli is a rare entity with only a handful of reported cases. It commonly presents as intractable slow transit constipation. We report a case of a one-year–old girl with chronic constipation that was subsequently diagnosed as desmosis coli, its clinical course and management with a review of the literature.

## Case summary

**Fig. 1:**
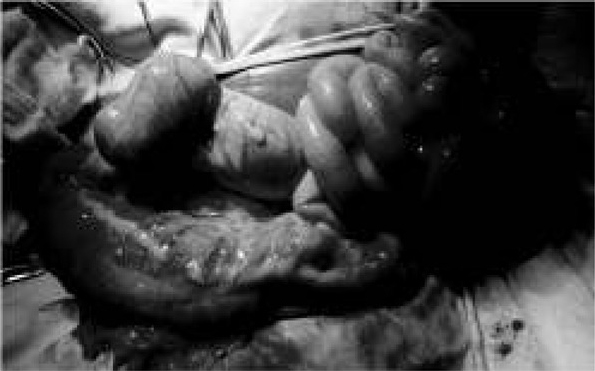
Grossly dilated colon transverse and sigmoid colon.

**Fig. 2:**
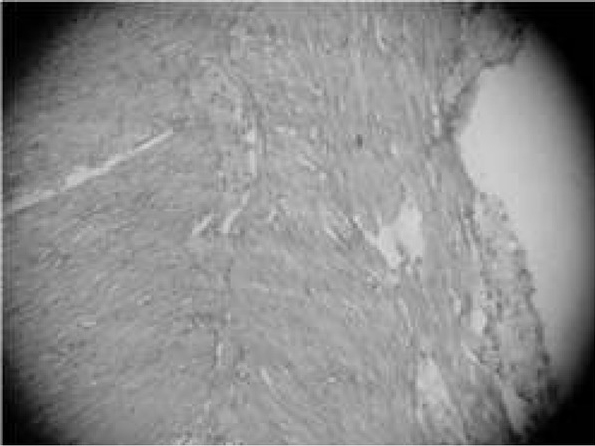
Lack of connective tissue between figure circular and longitudinal layer.

A 14-month-old female, hailing from western India, with a history of chronic constipation, presented with acute massive abdominal distension and vomiting. At laparotomy, a hugely dilated transverse and sigmoid colon with a transition zone at the lower sigmoid was found (fig. 1). Transverse stoma was done after taking multiple seromuscular biopsies. The patient underwent re-exploration on day 14 because of the non-functioning of the stoma and a fixed bowel loop. The histopathology report was suggestive of normal ganglion cells. Unfortunately the stoma continued not to function. A dye study showed dye in the colon after 24 hours ruling out any anatomical obstruction. Histopathology slides were reviewed multiple times and reported as lack of connective tissue of the colonic wall leading to the diagnosis of desmosis coli (fig. 2). The patient was started on gradual feeds and pro-kinetics and over the next 2 weeks the stoma slowly started functioning.

## Discussion

Constipation is a problem commonly encountered by paediatric surgeons all over the world. Although the exact incidence of constipation in Indian children is not known, according to a systematic review, world-wide incidence is 0.7% to 29% [1]. The causes of constipation are varied and the treatment can at times be frustrating. The most common cause of constipation is idiopathic or functional [2], however, there are various other causes like dietary factors, poor toilet training/habits, medical causes like hypothyroidism, hypocalcaemia etc, surgical causes like Hirschsprung’s disease, anorectal malformations, neural tube defects, visceral myopathies. Amongst all these disorders visceral myopathies are seldom encountered (0.7%) [2] and rarely diagnosed. Desmosis coli is an extremely rare cause of slow transit constipation. Due to its rarity, there is a lack of experience amongst paediatric surgeons in managing this entity. Hence, we would like to discuss this case with a review of the literature.

The term *desmosis coli* was introduced by Meier-Ruge, for a special pattern of structural abnormalities of the intestinal wall [3]. Partial or complete lack of the collagen mesh network was found in the biopsy specimens of the intestinal wall in 14 patients. Either dysganglionosis or hypoganglionisis was found, but aganglionosis was never found [4]. Two subtypes have been described: congenital primary type (rare) which is also known as aplastic desmosis, and secondary adulthood onset (relatively commoner), also known as atrophic desmosis [5]. The connective tissue mesh network has an active function in co-ordinated contractile peristaltic activity. Contraction of the circular muscle synchronously relaxes the longitudinal muscle [4]. Meier-Ruge has hypothesized that the lack of continuity of this mesh network abolishes the co-ordinated propulsive activity in the colon leading to therapy-resistant chronic constipation with hypoperistalsis or aperistalsis [6]. Primary desmosis of the colon, as the name implies, is congenital, whereas focal atrophic desmosis is generally seen as secondary to diverticulitis, tumor irradiation, Crohn’s disease, necrotizing enterocolitis, etc [7].

A Pubmed and Dynamed database search was done using the term “Desmosis Coli”: there are only about five reported cases of desmosis coli. In a series of four cases published by Hubner & Meier-Ruge, the commonest presenting feature was late-onset constipation with encopresis. The patients underwent multiple therapies in the form of laxatives, enemas, colostomies, which were futile. Ultimately all patients were diagnosed by seromuscular biopsies to be cases of desmosis coli that were subsequently treated by partial or total colectomy [3]. Similarly in the case discussed above, the patient presented with late-onset constipation and was initially managed as a case of Hirschsprung’s disease, but subsequently a review of histopathology diagnosed the case as desmosis coli. In a large series of 236 patients treated surgically for constipation, the authors found 14 (6%) cases of desmosis coli confirmed by the histopathology of the resected specimen. In another series published by Donald Marshall and Meier-Ruge, the authors report an association between Hirschsprung’s disease and desmosis coli.

Histopathologically desmosis coli is characterized by loss of tendinous structures in circular and longitudinal muscles and a loss of the myenteric tendinous plexus fascia which abolish directed peristalsis [8]. Similarly, in the case discussed above, the histopathology was consistent with such a description.

To conclude, desmosis coli is a rare cause of constipation, which should be suspected in cases where aganglionosis has been ruled out, and the constipation is refractory to conventional therapy. Pathologists should be aware of this condition, which will help in the diagnosis. The treatment of desmosis coli is controversial and should preferably be tailored to suit the patient. Patients should be counseled regarding the nature of treatment, prognosis and the possibility of a permanent stoma.
